# Comparative study on drug encapsulation and release kinetics in extracellular vesicles loaded with snake venom L - amino acid oxidase

**DOI:** 10.1186/s40360-025-00938-8

**Published:** 2025-05-08

**Authors:** Divya Ramesh, Shankar M. Bakkannavar, Vinutha R. Bhat, K. Sreedhara Ranganath Pai, Krishna Sharan

**Affiliations:** 1 Department of Forensic Medicine and Toxicology, Kasturba Medical College, Manipal, Manipal Academy of Higher Education, Manipal, Karnataka 576104 India; 2 Department of Biochemistry, Kasturba Medical College, Manipal, Manipal Academy of Higher Education, Manipal, Karnataka 576104 India; 3 Department of Pharmacology, Manipal College of Pharmaceutical Sciences, Manipal, Manipal Academy of Higher Education, Manipal, Karnataka 576104 India; 4 Department of Radiotherapy, K S Hegde Medical College, K S Hegde Medical Academy Mangalore, Mangaluru, 575018 India

**Keywords:** Extracellular vesicles, Snake venom L amino acid oxidase, Encapsulation efficiency, Drug delivery, Nanoparticles

## Abstract

**Background:**

This study aimed to evaluate the potential of plasma-derived extracellular vesicles (EVs) as drug delivery carriers by employing two drug-loading techniques: coincubation and freeze–thaw cycles.

**Methods:**

EVs isolated via the polyethylene glycol (PEG) precipitation method were characterized via nanoparticle tracking analysis (NTA) and transmission electron microscopy (TEM). The size of the particles was 200.1 ± 66.6 nm. The isolated vesicles were loaded with 1000 µg/ml snake venom L amino acid oxidase (SVLAAO) via the coincubation method and subjected to freeze–thaw cycles to prepare a novel formulation. The encapsulation efficiency (EE) of the loaded EVs was analysed at 30 and 60 min, and in vitro drug release profiles were evaluated for both methods and kinetic model for the same was determined.

**Results:**

The coincubation method achieved an EE of 58.08 ± 0.060% after 60 min, which was greater than that of the freeze–thaw method (55.80 ± 0.060%). Drug release studies demonstrated that 93% of the drug was released in 8.5 h by the coincubation method, whereas the freeze–thaw method resulted in faster release (99% in 6.5 h) due to membrane disruption. The best fit value (R^2^) was highest for zero order kinetics model.

**Conclusion:**

In conclusion, the coincubation method preserves EV membrane integrity, enabling sustained drug release, making it a promising strategy for targeted drug delivery applications. This study highlights plasma-derived EVs as innovative carriers for therapeutic delivery.

**Graphical Abstract:**

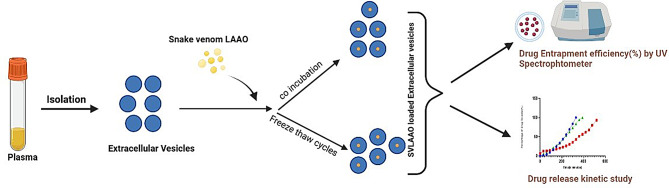

**Supplementary Information:**

The online version contains supplementary material available at 10.1186/s40360-025-00938-8.

## Introduction

Drug delivery involves the formulation of pharmacologically active substances for desired therapeutic outcomes when these substances are administered. Hence, it has been considered a fast-emerging field in nanoscience in recent decades [1]. Although there has been a marked increase in the development of nano delivery tools, certain disadvantages exist, such as off-target delivery, lower efficacy, and reactions with other substances, thus making the system less effective [2]. Thus, there is a need to develop a novel, innovative drug delivery system that increases the efficacy of the reaction, reduces the off-target distribution and causes the controlled release of the drug.

In recent years, the development of synthetic nanocarriers for drug delivery has tremendously increased. Liposomes, dendrimers, micelles, nanocapsules, etc., constitute the most common types of synthetic drug carriers [3]. Among these, liposomes, are considered the most versatile and are used in various nanoformulations [4]. Despite their numerous advantages, and its therapeutic efficiency, there are many disadvantages, such as off-target delivery, increased clearance from the reticuloendothelial system, and increased accumulation in the liver and other organs, thus limiting its availability in the target site.

In addition to lipid-based carriers, other nanocarriers called extracellular vesicles (EVs) derived from cells have gained increased importance in the last few years. EVs are tiny lipid-bound particles that are important regulators of many pathophysiological reactions. Recent studies explain the similarities between liposomes and EVs as drug delivery systems and the presence of phospholipids. However, the difference lies in the specific key markers on the EVs, which aid in targeting specific areas [5, 6].

EVs are classified into different types, such as ectosomes, exosomes, microvesicles, and apoptotic bodies. Although EVs are derived from different sources, they are classified according to their biogenesis pathway [7]. Exosomes are specific intraluminal vesicles formed during the maturation and development of multivesicular endosomes. The size of the exosomes is generally less than 200 nm. These tiny vesicles contain many surface markers, such as CD9, CD37 and CD63, which help with protein translocation and sorting [8–10]. Microvesicles or microparticles are EVs originating from the cell outer membrane. Apoptotic bodies (ApoBDs) are EVs released by cells undergoing programmed cell death or apoptosis.

EVs are present in almost all biological fluids, such as blood plasma, serum, urine, breast milk, and amniotic fluid [11]. The most commonly used source for EV biosynthesis is blood plasma, which is the most stable and biocompatible with the body’s physiological conditions. Plasma-derived EVs enhance the transport of drugs through biological barriers [12]. For these reasons, plasma-derived EVs are considered the best vehicles for cargo transportation. In some instances, plasma-derived EVs are involved in several pathological conditions and, hence, can be used as biomarkers for various diseases [13]. Hence, EVs derived from cells or any biological fluid can be used as therapeutic agents by loading the necessary drugs into them and targeting the same site of interest.

EVs are believed to be involved in important physiological processes, such as the differentiation of cells [14]. The primary functions of EVs include transporting various substances to target organs. These vesicles act as vehicles to transport substances such as RNA species, DNA and various proteins [15]. All these properties of EVs make them among the most efficient and versatile drug carriers.

The loading of the EVs is based on the properties of the cargo to be loaded. Passive loading is the easiest method for loading drugs into EVs. This phenomenon occurs when the cells are cocultured with the drug of interest (cargo to be loaded) for a specific time. In this method, the cells incorporate the cargo and package it into the EVs during the latter formation. As this method is simple to perform, the loading efficiency is very low compared with that of active loading. Moreover, it is difficult to control the amount of drug loaded.

The passive incubation method involves coincubation of the drug/cargo to be loaded with the EVs at room temperature for a specified period (usually 30 min to 60 min). Compared with the other techniques, coincubation is the most straightforward loading strategy. Passive loading occurs when the cargo concentration in the outer environment is high. Whereas Active loading occurs against the concentration gradient and results in the formation of pores in the EV membrane, thus resulting in efficient loading [16]. The active method involves loading the cargo into the EV by disrupting its membrane [17]. The active loading methods include electroporation, sonication, saponin treatment, freeze‒thaw cycles, etc. Compared to higher loading efficiency of electroporation and sonication, freeze-thaw cycles have intermediate loading efficiency.

Loading by freeze thaw cycles provide intermediate loading efficiency that involves disruption of the EV membrane by rapid freezing (-80 °C) and thawing at room temperature (37 °C) for a specified amount of time. This method is repeated for three continuous cycles so that the EV membrane is damaged, and the cargo is loaded successfully [18]. Here, membrane damage is not permanent and occurs due to the formation of ice crystals on the membrane so that the water-soluble cargo can enter the EVs [19]. Thus, compared with passive loading techniques, active loading is complex, as it involves a risk of rapid EV membrane disruption, which may damage the functions of EVs [20].

The choice of loading method depends on the characteristics of the drug to be loaded, the physiological conditions under which the drug must act, and the desired therapeutic efficiency of the cargo [21]. The optimum method for drug loading should provide maximum loading efficiency with less damage to the EV.

This research highlights the potential of plasma-derived EVs as effective drug carriers. This study emphasized the loading of snake venom-derived L amino acid oxidase (SVLAAO) into extracellular vesicles isolated from plasma. SVLAAO has emerged as a potential therapeutic since it possesses antimicrobial and anticancer properties. LAAO from Bathrops snake venom can inhibit biofilm formation by *Escherichia coli* and *Staphylococcus aureus* by altering the morphology of these species, resulting in antimicrobial properties [22]. Several other studies have shown that SVLAAO can induce cell death, thus inhibiting cancer cell metastasis [23]. This property of the snake venom L amino acid oxidase makes it potentially therapeutic against cancer.

This study involves loading snake venom-derived L-amino acid oxidase into plasma-derived extracellular vesicles via two different methods: coincubation and freeze‒thaw cycles. Because of its proven cytotoxic and apoptotic effects on the C6 rat glioma cell line in vitro, snake venom L-amino acid oxidase (SV-LAAO) was selected as the appropriate drug to be loaded into extracellular vesicles (EVs). This discovery, which was made possible by a pilot study, offered a strong rationale for additional research into the possible therapeutic use of SV-LAAO-loaded EVs in the treatment of glioblastoma.

The outcome was evaluated via encapsulation efficiency and drug release studies. Moreover, this study evaluates the impact of two different loading techniques into the human plasma derived extracellular vesicles and was evaluated by the extent of the drug release, thus checking the efficiency of human plasma derived EVs as a potent drug carrier that will be targeted to glioblastoma multiforme (brain tumour) in vivo (Wistar rats).

## Materials and methods

### Materials

**Fig. 1:**
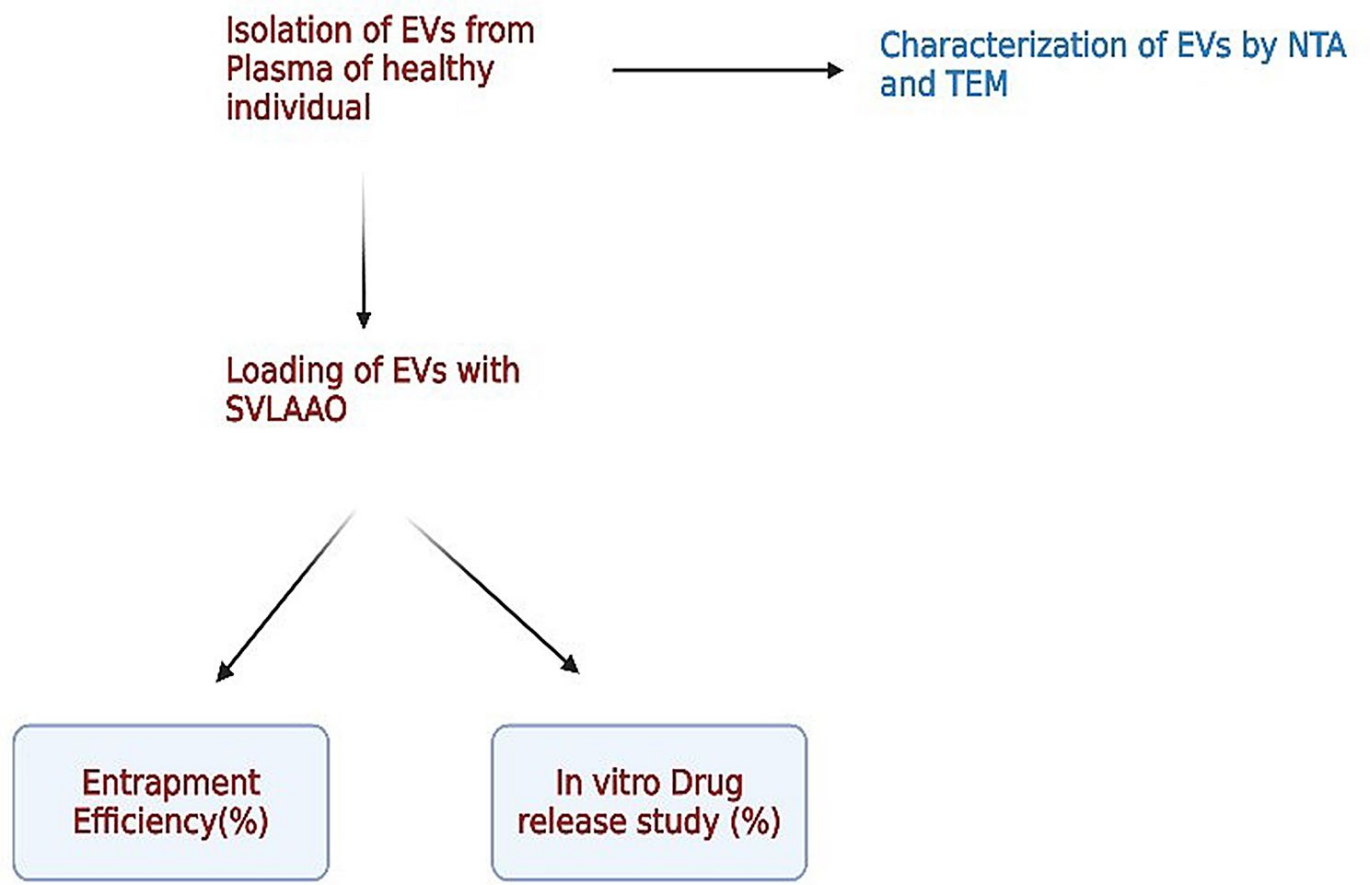
Schematic representation of the methodology (created with BioRender.com)

Snake venom L amino acid oxidase was purchased from Sigma Aldrich (Cat no: A5147); human blood samples were collected from healthy volunteers after institutional ethical approval was obtained from Kasturba Hospital Manipal (IEC no: 68/2022). Institutional biosafety committee clearance products were obtained from the School of Life Sciences Manipal in August 2022, including polyethylene glycol (w/v, 6000, Sigma‒Aldrich 81260), phosphate buffer saline (pH 7.4,6.4), and dialysis membranes. A schematic representation of the methodology is depicted in Fig. 1.

### Methods

#### Isolation of extracellular vesicles from human plasma

Extracellular vesicles were isolated from the plasma of healthy individuals via the polyethylene glycol precipitation method [24, 25]. Informed consent was obtained from all individual participants included in the study. Five milliliters of blood was collected from healthy individuals in an EDTA-coated vacutainer, and the plasma was separated by centrifugation at 2500xg for 20 min. The separated plasma was centrifuged at 2500xg for 20 min to remove RBCs and other debris. The collected supernatant was treated with 2 × 12% polyethylene glycol (PEG) and incubated overnight at 4 °C. The mixture was centrifuged at 3300xg for 1 h at 4 °C to separate the pellet and the supernatant. The resulting pellet was resuspended in sterile PBS (1–3 ml), an equal amount of 2 × 5% PEG was added, and the mixture was incubated at 4 °C for 1 h. In the final step, the mixture was centrifuged at 3300xg for 1 h at 4 °C, and the obtained pellet (EVs) was resuspended in 200 µl of sterile PBS (pH 7.4).

#### Characterization of extracellular vesicles

The plasma-derived EVs were characterized according to the MISEV 2023 guidelines [26]. The concentration of the isolated EVs and their size were determined via nanoparticle tracking analysis (NTA) via a NanoSight LM10 (Malvern Instruments, UK). EV samples resuspended in PBS were vortexed and diluted 1:1000 to detect particles within the optimum detection limit. The experiments were performed three times to identify and confirm the EVs isolated from the plasma.

##### Morphology of the EVs

The structure of the EVs was determined via transmission electron microscopy (TEM). TEM was performed via the negative staining method. The isolated plasma-derived EVs were diluted 1:5, and the suspensions were applied to carbon-coated grids at room temperature. Carbon-coated grids were stained with 0.5% uranyl acetate and dried at room temperature in the air. The negatively stained images were captured with a 120 kV JEOL multipurpose analytical transmission electron microscope.

#### Drug loading into plasma-derived extracellular vesicles Preparation of the formulation

The drug of interest, snake venom L amino acid oxidase (SV LAAO), was loaded into the plasma-derived EVs by coincubation of the SVLAAO with the EVs and freeze‒thaw cycles.

##### Coincubation method

For the coincubation method, 1000 µg/ml SVLAAO was coincubated with the EVs at room temperature for both 30 min and 60 min at a 1:1 ratio. The formulation was treated with an equal volume of 10% PEG and incubated at 4 °C for 1 h. The formulation was centrifuged at 10000 rpm for 1 h at 4 °C to separate the unentrapped drug. The results after 30 and 60 min of incubation were compared [27].

##### Freeze‒thaw cycle method

Drug loading was performed via the freeze‒thaw method by adding an SVLAAO concentration of 1000 µg/ml to equal amounts of EVs at a 1:1 ratio. The mixture was incubated at room temperature for 30 min, followed by freezing at -80 °C for 30 min. Three freeze‒thaw cycles were performed to load the drug into the EVs successfully. The formulation was treated with an equal volume of 10% PEG, followed by incubation at 4 °C for 1 h. The formulation was centrifuged at 10,000 rpm for 1 h at 4 °C to separate the unentrapped drug [27].

#### Drug loading studies of SVLAAO-loaded extracellular vesicles

The absorbance (λmax) of SVLAAO was determined via a UV spectrophotometer. The absorbance limit was set in the range of 200–600 nm. The standard graph for SVLAAO was generated by preparing various standards of SVLAAO in the range of 1 µg/ml to 500 µg/ml. The absorbance peak of SVLAAO was determined, and the drug loading efficiency of the drug-loaded SVLAAO was determined via an indirect method by measuring the free unentrapped drug in the supernatant [28]. The concentration of the free drug was determined via a UV‒visible spectrophotometer at 278 nm. The encapsulation efficiency of the EVs was calculated via the following equation:

$$\begin{aligned}&{\mathbf{Encapsulation}}\:{\mathbf{Efficiency}}\:\left( {{\mathbf{EE}}} \right)\%\cr&\quad=\frac{\begin{aligned}&{\mathbf{Total}}\:{\mathbf{amount}}\:{\mathbf{of}}\:{\mathbf{drug}}\:{\mathbf{added}}\:{\mathbf{to}}\:{\mathbf{the}}\:{\mathbf{EV}} \\& \quad - {\mathbf{Amount}}\:{\mathbf{of}}\:{\mathbf{drug}}\:{\mathbf{in}}\:{\mathbf{the}}\:{\mathbf{supernatant}} \\ \end{aligned} }{{{\mathbf{Total}}\:{\mathbf{amount}}\:{\mathbf{of}}\:{\mathbf{drug}}\:{\mathbf{added}}\:{\mathbf{to}}\:{\mathbf{the}}\:{\mathbf{EVs}}}}\: \times \:100\: \\ \end{aligned} $$

#### Drug release study of drug-encapsulated extracellular vesicles

The solutions of snake venom LAAO (2 mg/ml) and EVs containing SVLAAO were taken in dialysis bags with a 12 kDa MWCO (1 mL equivalent to 2 mg/mL) and kept in 20 mL release medium containing PBS (pH = 6.4). After the beakers containing the release medium and magnetic beads were placed on magnetic stirrers at 100 rpm, the dialysis bags containing the drugs and formulations were placed in the release medium, 800 µL of each sample was collected and replaced with the release medium every 30 min for 24 h, and the samples were analysed via a UV spectrophotometer at 278 nm. The concentration of each sample was calculated via a standard plot, and a release pattern graph was plotted. The data obtained from the release study were tabulated and analysed with GraphPad Prism 8.0 software. In addition to the cumulative percentage of drug release, four models of release kinetics (zero order model, first order model, Higuchi model and Korsmeyer–Peppas) were used to fit experimental data obtained from the studies. The coefficient of determination(R^2^) values was used to select the model with better fitting for the experimental result.

### Statistical analysis

The results were analysed via GraphPad Prism version 8.0. All the data were analysed according to the Mean ± SEM. All the data were determined by paired t test and 2-way ANOVA. Values with the values *P* < 0.0001 were considered as significant. All experiments were conducted as *n* = 3.

## Results

### Characterization of the extracellular vesicles

**Fig. 2:**
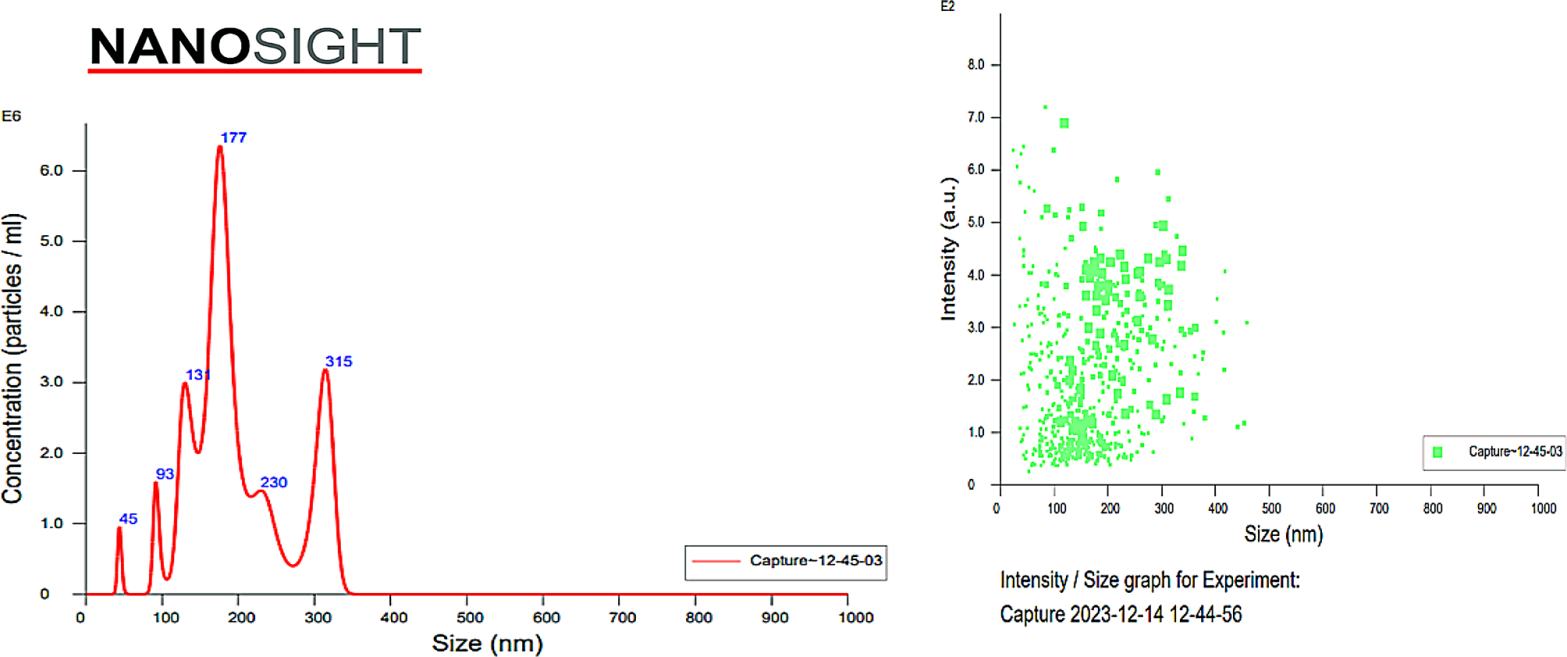
Depicts the results of the nanoparticle tracking analysis of the plasma-derived EVs. The concentration and particle size are shown in the figure

**Fig. 3:**
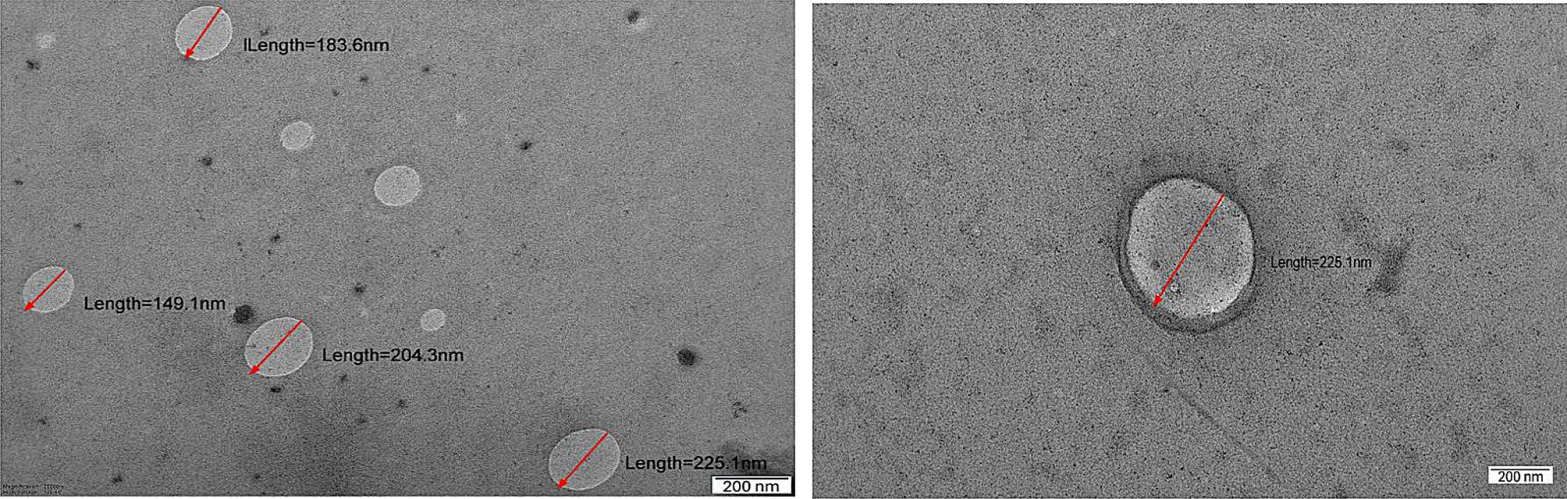
Transmission electron microscopy (TEM) image of the morphology of the EVs. The average size of the particles appears to be < 200 nm. Scaling done with ImageJ Software

Extracellular vesicles isolated from plasma via the polyethylene glycol precipitation (PEG) method were characterized for size via NTA, which revealed that the EVs had a mean size of 200.1 ± 66.6 nm and that the mode of the EVs was 176.1 nm (Fig. 2). The concentration of the particles was found to be 4.99 × 10^8^ particles/ml. The data were analysed via NanoSight software with a detection threshold of 5. Evaluation of the ultrastructure of the isolated EVs via transmission electron microscopy (TEM) with negative staining revealed the presence of many oval or circular double-membrane structures whose average size was < 200 nm as depicted in Table 1; Fig. 3.

**Table 1:** Table depicting the size of the Evs determined by the image J software

	Label	Area	Mean	StdDev	Length
1		112.594	160.903	22.126	183.662
2		125.312	143.928	19.855	204.391
3		91.646	161.623	25.913	149.132
4		138.03	132.22	18.098	225.125
5	Mean	116.896	149.668	21.498	190.578
6	SD	19.778	14.219	3.374	32.403
7	Min	91.646	132.22	18.098	149.132
8	Max	138.03	161.623	25.913	225.125

### Drug loading studies

**Fig. 4:**
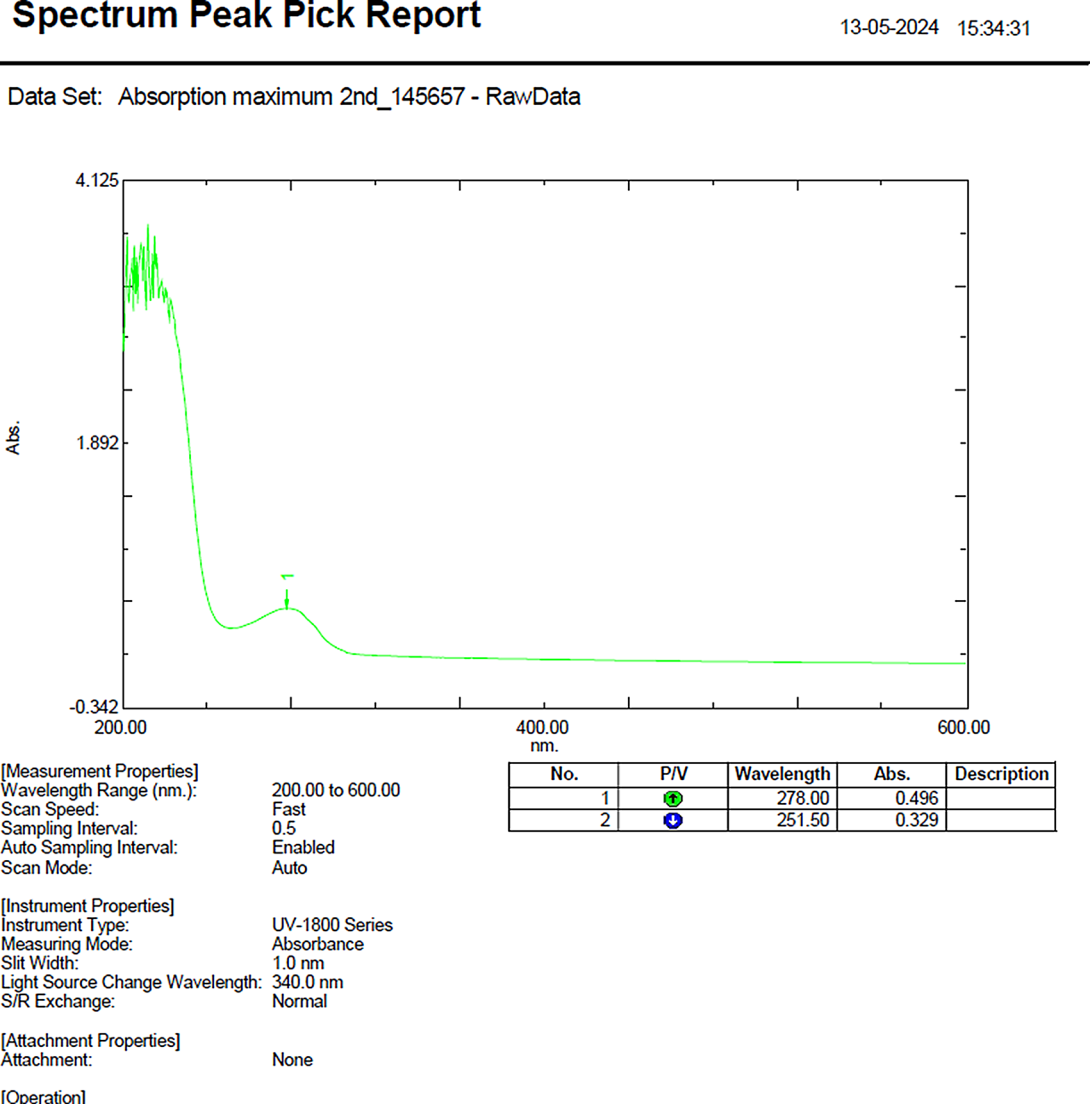
Spectrum peak pick report of SVLAAO

The absorbance (λmax) of SVLAAO was checked via a UV visible spectrophotometer in the range of 200–600 nm, and the maximum absorbance was found to be 278 nm. All further experiments were performed with a UV‒visible spectrophotometer using the obtained absorbance. The spectrum peak of SVLAAO is shown in Fig. 4.

#### Entrapment efficiency (EE) of the EVs

The entrapment efficiency (EE) of SVLAAO-loaded EVs by the coincubation method at a 1000 µg/ml concentration for 30 min and 60 min. The entrapment efficiency was greater at 60 min than at 30 min. The entrapment efficiency of the formulation was 26 ± 0.060 and 58.08 ± 0.060 at 30 min and 60 min of incubation, respectively. This indicated that approximately 58% of the drug was entrapped in the plasma-derived EVs by the coincubation method for 60 min, and 26% of the drug was entrapped by coincubation for 30 min. The results after 30 min and 60 min of incubation were compared.

**Fig. 5:**
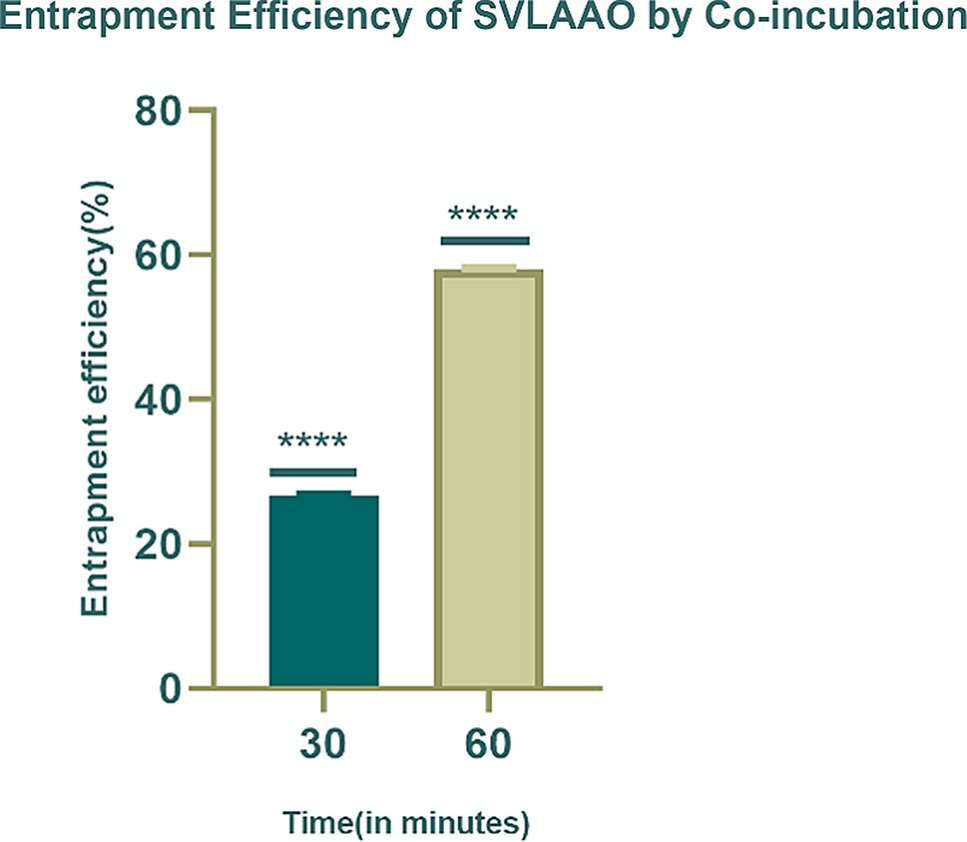
Graph showing the percentage entrapment efficiency (EE %) of EVs loaded with SVLAAO by the coincubation method for 30 min and 60 min. The graphs were drawn via GraphPad Prism 8. The graph shows that more drug was entrapped over a more extended period of incubation

Table 2 below depicts the entrapment efficiency of the EVs by coincubation methods at 30 min and 60 min. A graph comparing the percentage of entrapment efficiency at two different time points is shown in Fig. 5.

**Table 2:** Table depicting the entrapment efficiency by the coincubation method

Sample concentration (µg/ml)	Abs278	Value (graph)	Dilution factor	Final Value	EE (%)	Mean ± SD (of replicates 1–3)
Drug Entrapment Efficiency by Coincubation Method (30 min)						
1000 (Replicate 1)	0.714	366.42	2	732.84	26.71	26.6807 ± 0.060774
1000 (Replicate 2)	0.714	366.42	2	732.84	26.71	
1000 (Replicate 3)	0.715	366.94	2	733.89	26.61	
Drug Entrapment Efficiency by Coincubation Method (60 min)						
1000 (Replicate 1)	0.416	209.57	2	419.15	58.08	58.15439 ± 0.060774
1000 (Replicate 2)	0.415	209.57	2	418.10	58.08	
1000 (Replicate 3)	0.415	209.05	2	418.10	58.18	

**Fig. 6:**
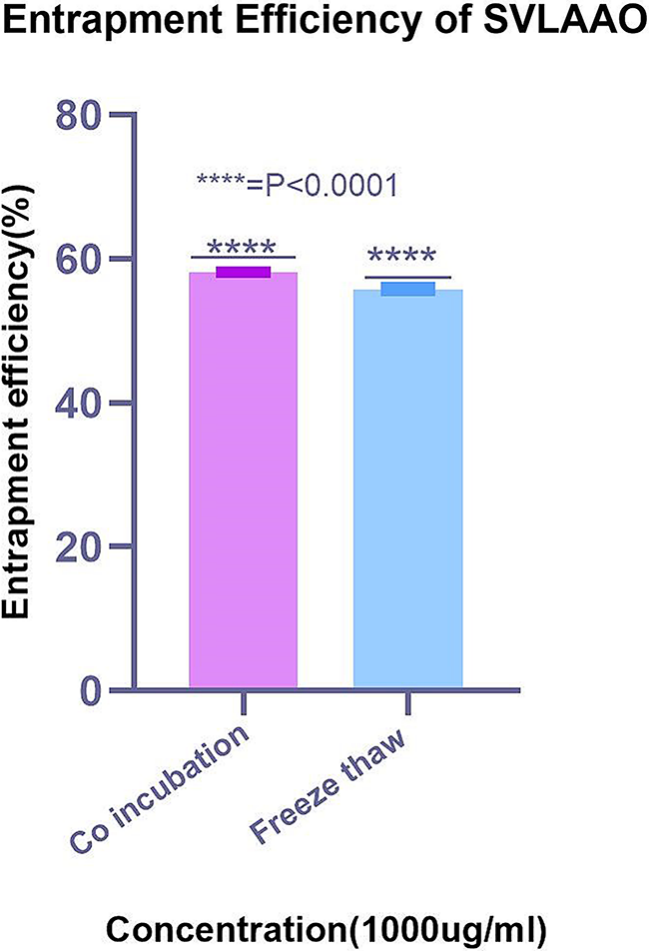
Graph showing the percentage entrapment efficiency of the coincubation and freeze‒thaw methods. The graphs were drawn via GraphPad Prism 8. The EE% of the Coincubation method is greater than that of the freeze‒thaw cycles

The entrapment efficiency of the EVs loaded with SVLAAO by coincubation was compared with the entrapment efficiency of EVs loaded with SVLAAO by freeze‒thaw cycles. The results indicated that the EV-loaded coincubation method resulted in greater entrapment than did the freeze‒thaw cycles. The percentage of entrapment by the incubation method was 58.5 ± 0.060774, whereas the percentage of entrapment by the freeze‒thaw method with the same drug concentration was 55.80 ± 0.060774. The results of the entrapment efficiency according to the number of freeze‒thaw cycles are shown below in Table 3. A comparison of the percentages of encapsulation efficiency of the two different methods is shown below in Fig. 6.

**Table 3:** Table depicting the entrapment efficiency by freeze‒thaw cycle

Sample concentration (µg/ml)	Abs278	Value (graph)	Dilution factor	Final Value	EE (%)	Mean ± SD (of replicates1-3)
**Drug Entrapment Efficiency by Freeze‒thaw cycle**						
1000 (Replicate 1)	0.438	221.15	2	442.31	55.76	55.80351 ± 0.060774
1000 (Replicate 2)	0.438	221.15	2	442.31	55.76	
1000 (Replicate 3)	0.437	220.63	2	441.26	55.87	

#### In vitro drug release study

**Fig. 7:**
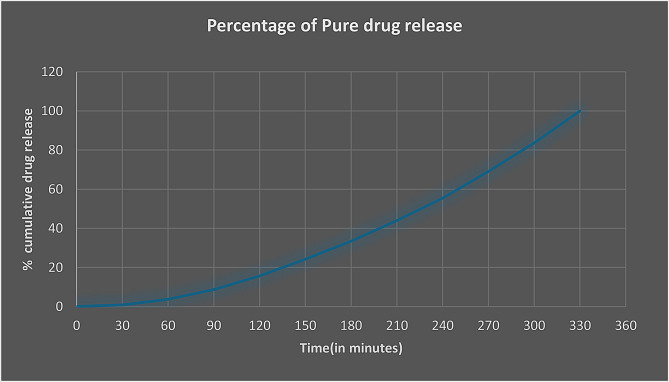
Graph showing the percentage of the pure drug (SVLAAO) released over time. The graph shows that approximately 99% of the SVLAAO was released into the medium within 5.5 h (330 min)

**Fig. 8:**
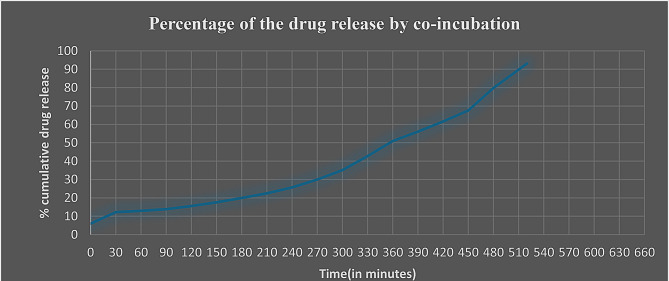
Graph showing the percentage of EV-loaded drug (SVLAAO) released by coincubation over time. The graph shows that approximately 93% of the SVLAAO was released into the medium within 8.5 h (510 min)

**Fig. 9:**
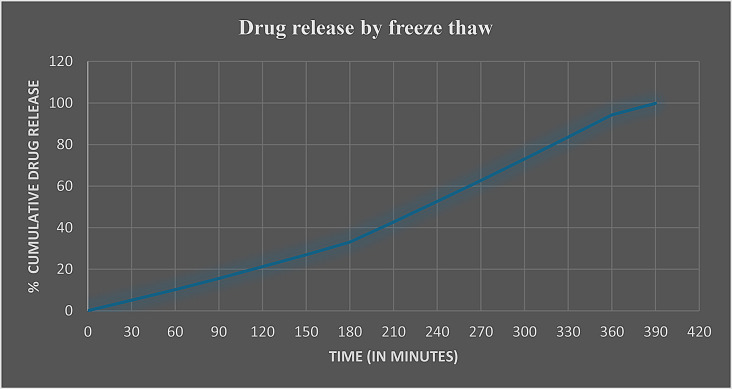
Graph showing the percentage of EV-loaded drug (SVLAAO) released by freeze‒thaw cycles over time. The graph shows that approximately 99% of the SVLAAO was released into the medium within 6.5 h (390 min)

**Fig. 10:**
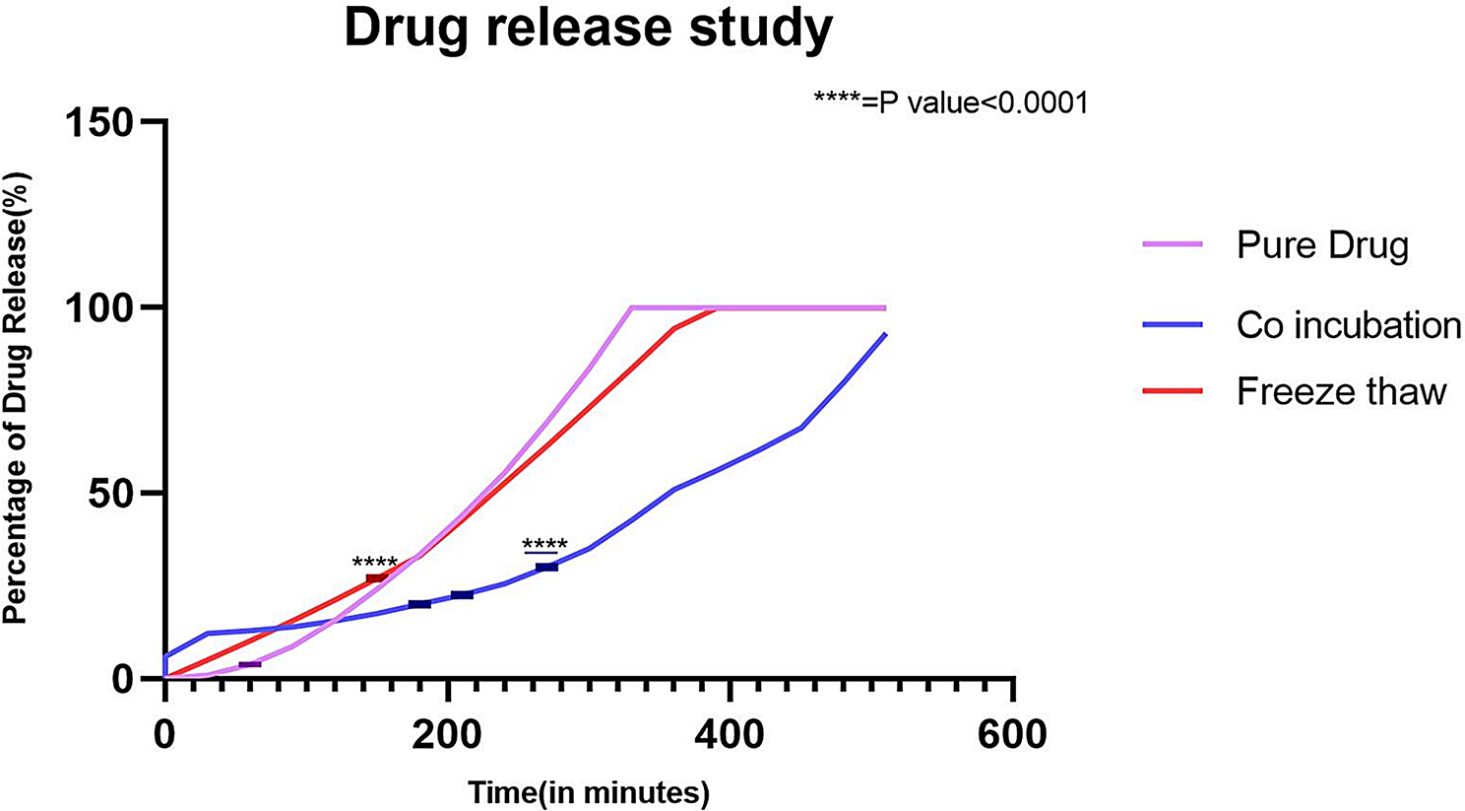
In vitro drug release of SVLAAO, SVLAAO-loaded EVs by coincubation and SVLAAO-loaded EVs by freeze‒thaw cycles at pH 6.4

In vitro drug release studies conducted by checking the drug release pattern from the SVLAAO-loaded EVs revealed the slow and sustained release of the drug into the medium for 24 h. 99% percent of the pure drug (nonencapsulated) was released into the medium over a period of 5.5 h, as shown in Table 4; Fig. 7. The drug encapsulated into the EV by the coincubation method was released into the medium in approximately 8.5 h, as shown in Table 5; Fig. 8, and the drug encapsulated by the freeze‒thaw cycles was released in 6.5 h, as shown in Table 6; Fig. 9. The results revealed that the drug encapsulated by the coincubation method exhibited slow and sustained release compared with the drug released by the freeze‒thaw cycle method, as shown in Fig. 10, thus indicating that the coincubation method is the most suitable technique for effective drug loading. The kinetic fit model was used to study the better fit data. Zero order model provided the better R value compared to first order, Higuchi and Korsmeyer–Peppas model, thus suggesting that the zero-order model fits perfectly for the following release study. The R^2^ values of all the models are depicted in Table 7.

**Table 4:** Drug release profile of the pure drug (SVLAAO)

Time (in minutes)	Percentage of the pure drug (SVLAAO) released
0	0.069 ± 0.0001
30	0.90 ± 0.0001
60	3.75 ± 0.0001
90	8.68 ± 0.0001
120	15.69 ± 0.0001
150	24.16 ± 0.0001
180	33.47 ± 0.0001
210	43.95 ± 0.0001
240	55.41 ± 0.0001
270	69.09 ± 0.0001
300	83.61 ± 0.0001
330	99.93 ± 0.0001

**Table 5:** Drug release profile of the Svlaao loaded extracellular vesicles (co-incubation)

Time (Minutes)	Release percentage (Coincubation)
0	5.97 ± 0.0001
30	12.22 ± 0.0001
60	12.98 ± 0.0001
90	13.88 ± 0.0001
120	15.55 ± 0.0001
150	17.56 ± 0.0001
180	20.00 ± 0.0001
210	22.50 ± 0.0001
240	25.62 + 0.0001
270	30.00 ± 0.0001
300	35.06 ± 0.0001
330	42.70 ± 0.0001
360	50.95 ± 0.0001
390	56.02 ± 0.0001
420	61.51 ± 0.0001
450	67.55 ± 0.0001
480	79.84 ± 0.0001
510	93.11 ± 0.0001

**Table 6:** Drug release profile of the SVLAAO loaded extracellular vesicles (freeze thaw)

Time (in minutes)	Release percentage (Freeze‒thaw)
0	0.055 ± 0.0001
30	5.05 ± 0.0001
60	10.22 ± 0.0001
90	15.61 ± 0.0001
120	21.22 ± 0.0001
150	27 ± 0.0001
180	33.05 ± 0.0001
210	42.72 ± 0.0001
240	52.66 ± 0.0001
270	62.72 ± 0.0001
300	73.05 ± 0.0001
330	83.61 ± 0.0001
360	94.33 ± 0.0001
390	99.83 ± 0.0001

**Table 7:** Table depicting the best fit kinetic models for release study

Group	Zero-order model (Q_t_ = Q_0_ + K_0_t)	First order model (ln Q_t_ = lnQ_0_ + K_1_t)	Higuchi model (Qt = K_H_ x t^1/2)^	Korsmeyer-Peppas model (Q_t_/Q_∞_ = K_k_t^*n*^)
Pure drug	R^2^ = 0.946	R^2^ = 0.457	R^2^ = 0.76	R^2^ = 0.946
Co-incubation	R^2^ = 0.916	R^2^ = 0.688	R^2^ = 0.75	R^2^ = 0.71
Freeze thaw	R^2^ = 0.980	R^2^ = 0.58	R^2^ = 0.84	R^2^ = 0.992

## Discussion

This study aimed to isolate extracellular vesicles (EVs) from human plasma and encapsulate SVLAAO by drug-loading methods involving coincubation and freeze‒thaw cycles. This study also investigated the release profile of the drug (SVLAAO) encapsulated in EVs via two different methods. In this study, EVs were isolated via the polyethylene glycol precipitation method. According to Otani et al. (2019), the extraction of extracellular vesicles with polyethylene glycol results in greater yields than the ultracentrifugation method does [29]. Zoia et al. (2022) isolated extracellular vesicles from the RBCs of human subjects via the polyethylene glycol precipitation method and reported that this method yielded greater amounts of EVs with sizes ranging from 100 to 300 nm [30].

In this study, SVLAAO was successfully loaded into plasma-derived EVs via coincubation and freeze‒thaw methods. The coincubation method involves loading drugs into EVs via incubation at room temperature for a specific period. The loading of drugs into EVs via the freeze‒thaw method involves repeated freeze‒thaw cycles lasting approximately 30 min each for three cycles. According to Shivakumar et al., 2023 coincubation method at room temperature is considered an apt method for loading, as it does not change or disrupt the membrane stability and integrity, thus maintaining the structure and properties of the EVs [27]. Gelibter et al. (2022) reported that exposure of EVs to freeze‒thaw cycles results in a reduction in the EV concentration and increased variability, thus reducing their therapeutic potential [31]. Moreover, treatment of EVs by freeze‒thaw cycles cause the loss of cargo, thus decreasing the potential to treat diseases [32].

The entrapment efficiency of SVLAAO was calculated via an indirect method in which the amount of unentrapped drug remaining in the supernatant at 278 nm was measured. According to Khalid et al., 2024, UV spectrophotometry is a non-destructive process that does not cause any structural damage to EVs, thus preserving the structure and functional integrity of EVs compared with other techniques [33]. According to Lowe et al.,2024 techniques such as high-performance liquid chromatography (HPLC) may lead to underestimation of the loading efficiency depending upon the different types of loading methods used [34]. Additionally, the variability of the EV particle size may complicate the process of checking the entrapment efficiency of the particles via HPLC. The percentage of the entrapment efficiency of the EVs loaded with SVLAAO by coincubation was greater than that of those loaded by the freeze‒thaw cycle. This result coincides with the study performed by Shivakumar et al. (2023), in which the entrapment efficiency was slightly greater for EVs loaded by coincubation than for EVs loaded by freeze‒thaw cycles [27]. This may be explained by the fact that slight membrane damage causes decreased drug loading compared with the coincubation method.

To analyse the amount of drug released over time, an in vitro drug release study was performed. Drug release from SVLAAO was fast, there was an immediate burst release, and 99% of the SVLAAO was released at 5.5 h. The SVLAAO-loaded EVs obtained via the coincubation method exhibited slow and sustained release of the encapsulated SVLAAO. Approximately 93% of the drug was released within 8.5 h. EVs loaded with SVLAAO via the freeze‒thaw method exhibited faster release of the drug than did those loaded via the former method. In this method, 99% of the drug was released in 6.5 h. These results align with those of Cheng et al. (2019), who reported that drug release from exosomes loaded with cargo via the freeze‒thaw method causes rapid release of the drug into the media due to alterations in the exosomal membrane [35].

Analysis of the kinetic release model for the drug release study was done to check the best fit model. The zero-order kinetic model exhibited R^2^ values of 0.946 for the release of the pure drug from the dialysis membrane, while R^2^ values of First order model, Higuchi model and Korsmeyer Peppas model were 0.457, 0.76 and 0.946 respectively. The R^2^ values of the EVs loaded by Co incubation method and Freeze thaw cycles also exhibited the similar trend. But the pure drug and the EVs loaded by the freeze thaw cycles exhibited higher R^2^ values in Korsmeyer Peppas model addition to the zero-order model, while the EVs loaded by the Co- Incubation method showed lower R^2^ values for the Korsmeyer Peppas model. This can be correlated with the study by Li et al.,2021 proved that the zero-order model enables the constant release of the drug over a period, hence this model provides the better therapeutic effect and lesser side effects [36]. But Ortiz et al., 2021, reported the best fit model for the drug release from the NLC (nanostructured lipid carrier) was Korsmeyer Peppas model [37]. Meanwhile, Woolfson et al.,2006 reported that the delivery of the hydrophobic drugs like TMC120 showed the zero-order kinetic model of release, with short initial period of the burst [38].

These results show that drug (SVLAAO) release was slow and sustained in the case of EVs loaded by the coincubation method, thus making it an apt method for drug loading, and the formulation created by this method may act as the best therapeutic agent that can be targeted to different target sites.

## Conclusion and future perspectives

In the current study, the potential drug-loading capacity of EVs isolated from the plasma of healthy individuals was determined. EVs isolated were characterized for specific characteristics such as morphology and size. The formulation was designed by loading SVLAAO into the EVs by two different methods (coincubation and freeze‒thaw cycles). The entrapment efficiency and the drug release study were performed with the formulation to confirm the effective loading method. Among the two loading methods, co-incubation of the EVs with SVLAAO for 60 min, exhibited higher percentage of entrapment compared to the freeze thaw cycles. The release kinetics model of this formulation indicated that EVs loaded via the coincubation, and freezing method released the drug in a controlled manner (zero order release kinetics) thus, making it a novel and promising formulation for therapeutic purposes.

In future studies, these nanovesicles can be used as carriers for various therapeutic agents and as the most effective therapeutics for various diseases, such as cancer and neurogenerative diseases. Since the stability of these nanovesicles is considered a limitation for their application, combining these naturally derived EVs with synthetic nanocarriers would produce more stable carriers with improved therapeutic capacity. The problem with off-target delivery can be overcome by using engineered EVs, wherein surface modification of EVs is performed to enhance their properties. The problem of large-scale production of EVs may be addressed by using larger bioreactors at lower costs at both the industrial and commercial levels. All these attempts may help create a bridge between the bench and the bedside, thus making extracellular vesicles successful therapeutic agent.

## Electronic supplementary material

Below is the link to the electronic supplementary material.

Supplementary Material 1

Supplementary Material 2

## Data Availability

Data is provided within the manuscript or supplementary information files.

## Abbreviations

SVLAAO: Snake venom L amino acid oxidase
LAAO: L -amino acid oxidase
EV: Extracellular vesicle
CD9, CD37, CD63: Cluster of differentiation 9,37,63
ApoBDs: Apoptotic bodies
PEG: Polyethylene glycol
NTA: Nanoparticle Tracking Analysis
TEM: Transmission Electron microscopy
EE: Entrapment Efficiency
