# Correction: Comparative study on drug encapsulation and release kinetics in extracellular vesicles loaded with snake venom L - amino acid oxidase

**DOI:** 10.1186/s40360-025-00956-6

**Published:** 2025-06-05

**Authors:** Divya Ramesh, Shankar M. Bakkannavar, Vinutha R. Bhat, K. Sreedhara Ranganath Pai, Krishna Sharan

**Affiliations:** 1 Department of Forensic Medicine and Toxicology, Kasturba Medical College, Manipal, Manipal Academy of Higher Education, Manipal, Karnataka, 576104 India; 2 Department of Biochemistry, Kasturba Medical College, Manipal, Manipal Academy of Higher Education, Manipal, Karnataka, 576104 India; 3 Department of Pharmacology, Manipal College of Pharmaceutical Sciences, Manipal, Manipal Academy of Higher Education, Manipal, Karnataka, 576104 India; 4 Department of Radiotherapy, K S Hegde Medical College, K S Hegde Medical Academy Mangalore, Mangaluru, 575018 India

**Correction: BMC Pharmacol Toxicol 26, 98 (2025)**

10.1186/s40360-025-00938-8

Following publication of the original article two errors concerning the authorship were reported.

The first error was that Vinutha R. Bhat was not marked as a corresponding author in the original publication. This Correction and the updated article now correctly list both Shankar M. Bakkannavar and Vinutha R. Bhat as corresponding author.

The second error is that in affiliations 1, 2 and 3 that ‘Manipal’ was missing following Kasturba Medical College and Manipal College of Pharmaceutical Sciences. The corrected affiliations are given below with the previously missing ‘Manipal’ highlighted in bold.

1 Department of Forensic Medicine and Toxicology, Kasturba Medical College, **Manipal**, Manipal Academy of Higher Education, Manipal, Karnataka 576104, India.

2 Department of Biochemistry, Kasturba Medical College, **Manipal**, Manipal Academy of Higher Education, Manipal, Karnataka 576104, India.

3 Department of Pharmacology, Manipal College of Pharmaceutical Sciences, **Manipal**, Manipal Academy of Higher Education, Manipal, Karnataka 576104, India.

The original article has been updated.

